# Generation of siRNA Nanosheets for Efficient RNA Interference

**DOI:** 10.1038/srep25146

**Published:** 2016-04-28

**Authors:** Hyejin Kim, Jae Sung Lee, Jong Bum Lee

**Affiliations:** 1 Department of Chemical Engineering, University of Seoul, Seoul 02504, Korea

## Abstract

After the discovery of small interference RNA (siRNA), nanostructured siRNA delivery systems have been introduced to achieve an efficient regulation of the target gene expression. Here we report a new siRNA-generating two dimensional nanostructure in a formation of nanosized sheet. Inspired by tunable mechanical and functional properties of the previously reported RNA membrane, siRNA nanosized sheets (siRNA-NS) with multiple Dicer cleavage sites were prepared. The siRNA-NS has two dimensional structure, providing a large surface area for Dicer to cleave the siRNA-NS for the generation of functional siRNAs. Furthermore, downregulation of the cellular target gene expression was achieved by delivery of siRNA-NS without chemical modification of RNA strands or conjugation to other substances.

Attention towards ribonucleic acid (RNA) has naturally grown due to its myriad of biological potentials. In addition to messenger RNA (mRNA) carrying genetic information within organisms, non-coding RNAs such as transfer RNA (tRNA), ribosomal RNA (rRNA), short-interfering RNA (siRNA) and small nuclear RNA (snRNA) can also serve particular roles in biological system^1^. In addition, RNA has been recently used as a generic material for nano- and micro-architectures in the scope of materials engineering to utilize a wide range of its functions^2^,^3^,^4^. In particular, after the discovery of RNA interference (RNAi) in 1998^5^, RNA architectures carrying Dicer substrates have been widely investigated. For Dicer enzyme to generate small interference RNA (siRNA) or micro RNA (miRNA) to induce gene silencing effect, RNA nanostructures have been designed to bear double-stranded RNA (dsRNA). With this consideration, various RNA structures were developed with the shapes of dumbbell^6^,^7^,^8^, branched multimer^9^ and dendrimer^10^. By rational design, siRNAs generated from the artificial RNA structures can inhibit expression of virtually any gene in target-specific manner^11^.

In particular, after the development of our self-assembled RNAi-inducing microsponges bearing multiple hairpin structures as siRNA precursors^12^, our group has intensively explored for controllable synthesis of RNAi-inducing microscopic constructs to achieve high therapeutic efficacy. In the synthesis of RNAi microsponge, enzymatic approach was adapted to generate multiple copies of long RNA strands as well as to induce self-assembly of the strands. Moreover, size-controlling of RNA nanoparticles was successfully demonstrated recently via adjusting the concentrations of RNA polymerase in enzymatic replication reaction^13^. Building on previous achievements, a novel approach to prepare RNA membrane without polymer supports or complexation was also recently introduced^14^. This was the first example of a macroscopic RNA membrane, which is composed of RNA strands and has tunable mechanical properties and biological functions. In this study, we also demonstrated successful release of chemical drugs from our RNA membranes. In addition, we found that two-dimensional structure of the membrane was advantageous for effective siRNA generation by Dicer.

Inspired by membrane’s structural property, here we report the preparation of siRNA nanosized sheet (siRNA-NS) with lateral dimensions of <500 nm. To boost therapeutic potential of two-dimensional RNA structure with fully exploiting its material properties, such as large surface area and tunable biological functions, our RNA membrane was embedded with siRNA strands and fractured into nanosheets via ultrasonication to induce RNAi in cytoplasm (Fig. 1). By controlling degree of base-pairing, only functional parts, potential siRNA, are designed to form duplexes via hybridization. In this design, we can eliminate undesired formation of short duplexes that are not functional (Fig. 1a). From this partially complementary RNA membrane with bubbles between potential siRNAs that is relatively thinner and flexible, we could easily generate siRNA-NS via ultrasonication.

## Results and Discussion

**Figure 1:**
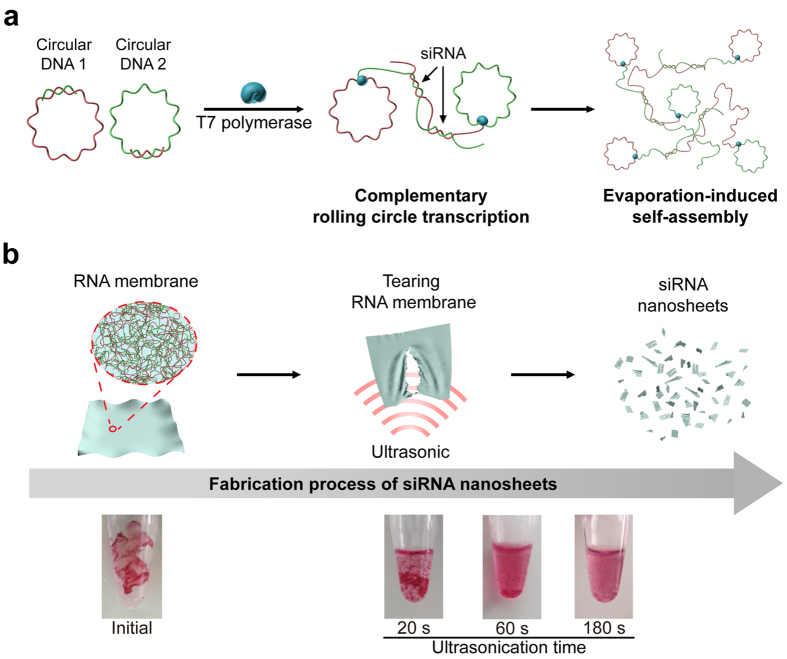
Schematic illustration of the synthetic process of siRNA-NS. (**a**) Design for siRNA-NS bearing siRNA precursors. Complementary rolling circle transcription (cRCT) is carried out by T7 RNA polymerase with circular DNA**1** and circular DNA**2**, partially complementary to circular DNA**1**. The cRCT process is followed by evaporation-induced self-assembly, completing the synthetic process of RNA membrane. (**b**) Schematic illustration and digital images of GelRed-stained RNA membrane after ultrasonication. As sonication proceeds, RNA membrane lost its initial form and torn into large pieces (~20 s). After 60 s of sonication, RNA membrane was broken into smaller pieces with large sediments still remaining. At the time point of 180 s after sonication, no sediment was observed with naked eye.

To synthesize siRNA-NS for mediating RNAi, two types of circular DNAs with rationally designed sequences were first generated using the method reported elsewhere^14^,^15^. Circular DNA1 and circular DNA2 depicted in Fig. 1a are rationally designed to encode sense strand and antisense strand for anti-GFP siRNA, respectively (Table 1). Similar to the method reported previously^14^, both circular DNAs bear promoter region for T7 RNA polymerase, thus two kinds of RNA strands can be generated repeatedly via complementary rolling circle transcription. By rational design, two partially complementary circular DNAs were used to generate repeating siRNA duplexes as fabrics for siRNA-NS. To be specific, several transcripts by RCT are hybridized and entangled with other neighbouring transcripts, then finally condensed into the membrane formulation through evaporation-induced self-assembly. By extensive ultrasonication, RNA membrane was easily torn up into the nanosized sheets (Fig. 1b). The digital camera image in Fig. 1b clearly shows the tearing process of RNA membrane to a small piece of membranes within several minutes of sonication.

**Figure 2:**
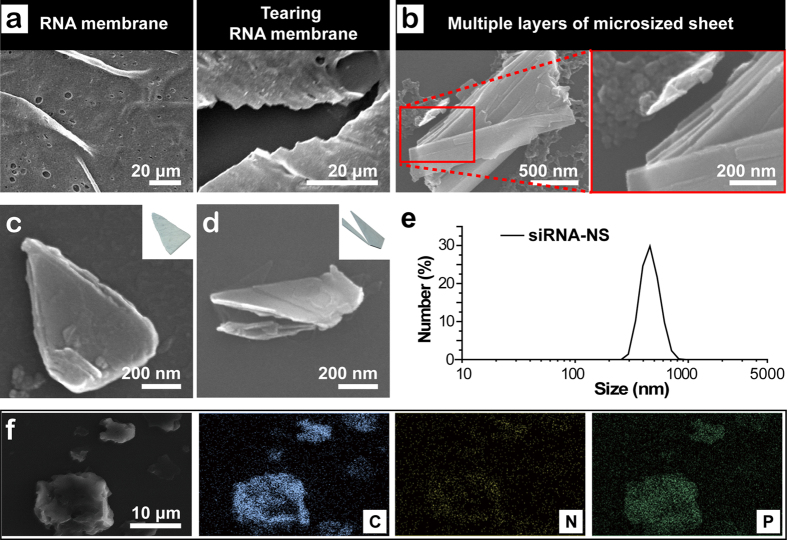
Characterization of the siRNA-NS. (**a**) SEM images of the surface and the site being torn of RNA membrane. (**b**) SEM images of the microsized RNA sheet after 10 min of sonication, and high magnification image of the highlighted area, revealing its multi-layered structure. (**c–d**) High magnification SEM images of siRNA-NS and schematic illustrations of each shape of the nanosheets (inset). (**e**) Size distribution of anti-GFP siRNA-NS obtained with dynamic light scattering analysis. (**f** ) SEM-based EDS mapping of siRNA-NS, revealing its composition.

To closely investigate the tearing process, each step of the process was observed by scanning electron microscope (SEM) including the image of intact RNA membrane (Fig. 2a). When introduced with ultrasonic pulses, RNA membrane starts to be torn in a similar way of a sheet of paper being torn. After a brief ultrasonication for 20 s, RNA membrane become fractured into large pieces still visible by naked eyes when stained with GelRed. After further sonication, dominating products were microsized (Fig. 2b). The high magnification SEM image of the split site of the RNA membrane in the process of production of siRNA-NS reveals that the RNA membrane is composed of multiple thinner layers. By ultrasonic impulses, the layers fall off from the membrane, and are simultaneously torn into small pieces. With further ultrasonication, sediments and floating pieces coexist in the reaction tube. After 20 min of vigorous sonication, the sheets finally become under 500 nm-sized, which is confirmed by SEM (Fig. 2c,d) and dynamic light scattering (DLS, Fig. 2e). As a result of sonication, morphology of siRNA-NS was varied. Further analysis with energy-dispersive X-ray spectroscopy (EDX) revealed siRNA-NS is composed of carbon (C), nitrogen (N), oxygen (O) and phosphorous (P), indicating siRNA-NS is made of RNA strands (Fig. 2f).

**Figure 3:**
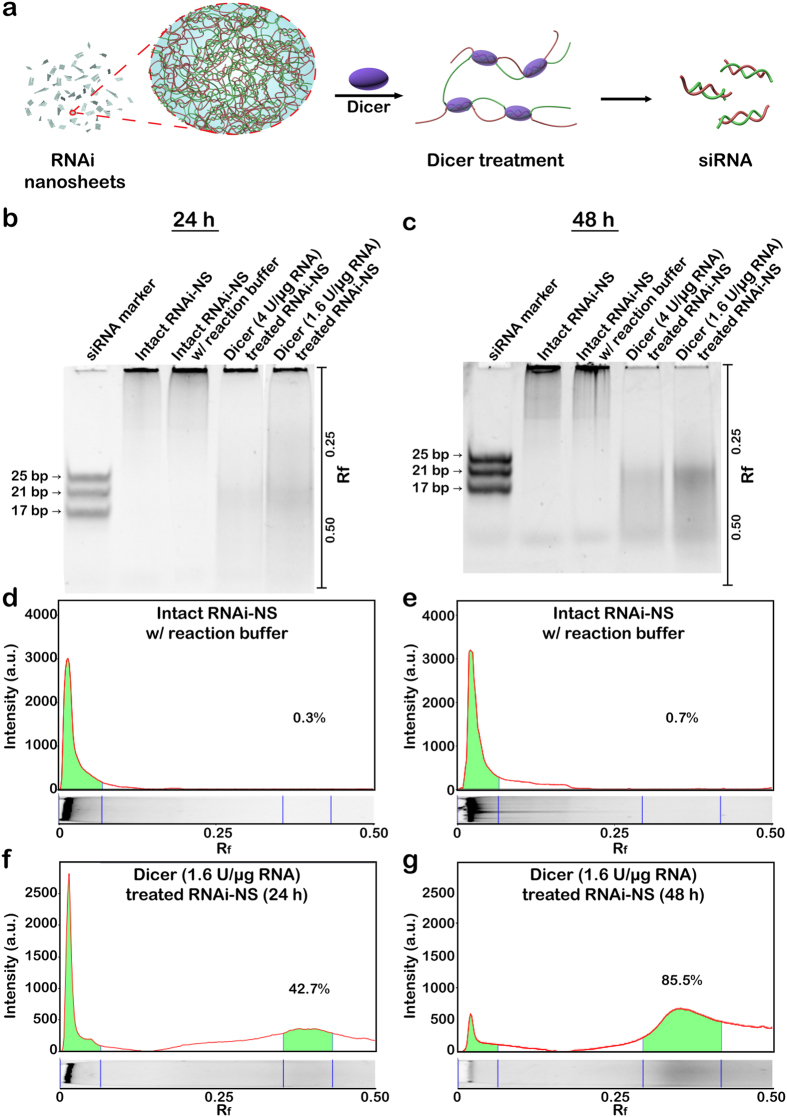
Synthesis of siRNA from siRNA-NS. (**a**) Schematic illustration of siRNA production from siRNA-NS. (**b,c**) Gel electrophoresis analysis of siRNA-NS treated with Dicer enzyme for 24 h (**b**) and 48 h (**c**). Lanes 1-3 indicate siRNA marker, untreated siRNA-NS (114 ng) and untreated siRNA-NS with Dicer reaction buffer, respectively. Lanes 4-5 indicate the same amount of siRNA-NS treated with 4 units or 1.6 units per μg RNA, respectively. (**d–g**) Band intensities of the lane 3 (**d,e**) and lane 5 (**f,g**) in each gel were analyzed. The values of R_f_ were indicated for the exact determination of the positions of the bands.

As illustrated in Fig. 3a, each siRNA-NS has numerous cleavage sites, and the cleaved product can later serve as functional siRNA. To examine the potential of siRNA-NS to generate embedded siRNAs, Dicer enzyme-mediated *in vitro* generation of siRNA duplexes was analysed by polyacrylamide gel electrophoresis (Fig. 3b,c). 42.7% of total RNA was cleaved to the length of ~23 bp by 24 h (Fig. 3f), and 85.5% of the total RNA was cleaved to the length of ~23 bp by 48 h (Fig. 3g). Compared to the micro-sized particle formulated short hairpin RNAs (shRNA) which was previously reported to generate 21% of cleavable siRNA strands^12^, siRNA-NS has shown capability of high-yield generation of siRNAs. This is because thin sheet-like 2-D structure of the siRNA-NS provides a large surface area for enzyme-substrate contact which enables the cleaving enzyme to work efficiently. However, biological stability of siRNA-NS was comparably higher than naked siRNA mainly due to densely packed internal structure of the nanosheets (see Supplementary Fig. S1).

**Figure 4:**
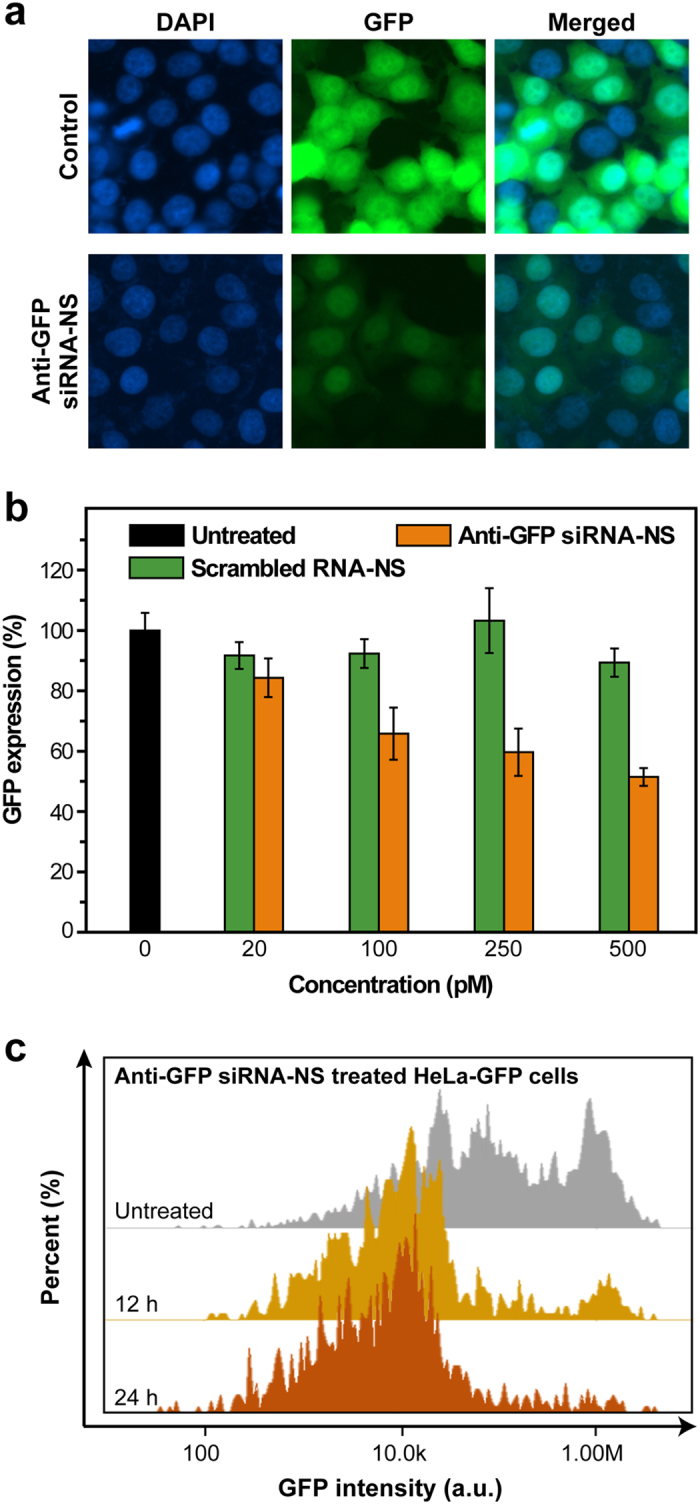
siRNA-NS-mediated gene knockdown assay. (**a**) Fluorescence microscopy images of HeLa-GFP cells at 24 h after treatment with 100 pM, 250 pM and 500 pM of anti-GFP siRNA-NS. Control cells were left untreated for 24 h. (**b**) GFP expressions of HeLa-GFP cells at 24 h after treatment with 20 pM, 100 pM, 250 pM and 500 pM of anti-GFP siRNA-NS (orange), scrambled siRNA-NS (green) or left untreated (black). GFP intensities were normalized with the intensity of untreated cells (n = 4). (**c**) Image cytometry analysis of anti-GFP siRNA-NS treated HeLa-GFP cells at 12 h (yellow) and 24 h (orange) after the treatment.

To examine therapeutic efficacy of siRNA-NS, HeLa cells expressing enhanced green fluorescence protein (HeLa-GFP) were treated with anti-GFP siRNA-NS. Decrease of green fluorescence intensities was confirmed at 24 h after the treatment of anti-GFP siRNA-NS, showing maximum silencing efficiency with 500 pM (Fig. 4a). The cellular GFP level of the anti-GFP siRNA-NS treated cells was reduced to 50% in 24 h, while scrambled siRNA-NS had negligible effect on the cellular GFP level (Fig. 4b). Cytometry analysis also presents green fluorescence intensities of the HeLa-GFP cells treated with anti-GFP siRNA-NS were reduced (Fig. 4c). Moreover, the target gene silencing effect of siRNA-NS persisted until 6 days post-transfection (see Supplementary Fig. S2), indicating a capability of long-term gene repression by delivering siRNA-NS.

In conclusion, nanosized RNA sheets were synthesized successfully with potential siRNA strands. For producing siRNA-NS, partially complementary RNA membrane which has 46 base-pairings out of 92 bases was chosen, so that one turn of T7 RNA polymerase around the circular DNA can result in two potential siRNA regions. Through repetitive polymerization on the template DNAs, long RNA strands can be generated, which results in multiple Dicer cleavage sites. In addition, this RNA membrane could be easily torn into nanosized sheets by ultrasonication because the membrane has flexible mechanical property. We have also demonstrated efficient generation of siRNAs from siRNA-NS in presence of Dicer enzyme and fast gene silencing effect in cells, which suggests siRNA-NS can be utilized as a new platform for efficient siRNA delivery. Therefore, over the advantage of using spherical structures, two dimensional nanosized RNA sheet could bolster therapeutic efficacy of RNA-based materials.

## Methods

### Materials and reagents

DNA oligonucleotides were purchased from Integrated DNA Technologies (IDT). T4 DNA ligase (Cat. No. M1804) was purchased from Promega. T7 RNA polymerase (Cat. No. M0251L), 10X RNA polymerase reaction buffer (Cat. No. B9012S) and ribonucleotide solution mix (Cat. No. N0466S) were purchased from New England BioLabs. GelRed (Cat. No. 41003) was purchased from Biotium. Dulbecco’s Modified Eagle’s Medium (DMEM, Cat. No. LM 001-10) and fetal bovine serum (FBS, Cat. No. S 101-01) were purchased from Welgene. 100 X Antibiotic-Antimycotic (Cat. No. 15240-062), trypsin-EDTA (Cat. No. R-001-100) and Dulbecco’s phosphate-buffered saline (DPBS, Cat. No. 14190-144) were purchased from Gibco. Transfection reagent (TransIT-X2 Dynamic Delivery System, Cat. No. MIR 6000) was purchased from Mirus.

### Circularization of linear DNA

Phosphorylated 92 base-long linear DNA and 22 base-long primer DNA at the final concentration of 10 μM were mixed in nuclease-free water. For denaturation and annealing, the mixture was heated at 95 °C for 2 min and slowly cooled to 25 °C for 1 h using thermal cycler (Bio-Rad). To ligate the nick in the circularized DNA, the solution was incubated overnight with 0.03 U μl^−1^ of T4 DNA ligase and ligase buffer (30 mM Tris-HCl (pH 7.8), 10 mM MgCl_2_, 10 mM DTT and 1 mM ATP).

### Generation of siRNA-NS

For complementary rolling circle transcription process, circularized DNA (final concentration of 3 μM) and complementary circular DNA (final concentration of 3 μM) were mixed with 15 mM of ribonucleotide solution mix, reaction buffer (60 mM Tris-HCl, 9 mM MgCl_2_, 3 mM spermidine and 1.5 mM dithiothreitol) and 5 U μl^−1^ of T7 RNA polymerase. The reaction solution was incubated for 20 h at 37 °C, then evaporation-induced self-assembly was carried out overnight^14^. After removal of the remaining solution, RNA membrane was washed three times with nuclease-free water. Then, RNA membrane was resuspended in 100 μl of nuclease-free water. The samples were placed on a water bath-type ultrasonicator, and ultrasonication pulses were applied to the samples for 20 min. The frequency and output of the sonication were 28 kHz and 50 watts, respectively, and the temperature was maintained at 4 °C. A small portion of micro-sized sheets were removed after centrifugation at 8000 rpm for 3 min.

### Characterization of siRNA-NS

S-5000 H (Hitachi) field emission scanning electron microscope (FE-SEM) was used to obtain high resolution images of siRNA-NS, and FE-SEM based energy dispersion X-ray (EDX) was used to analyse chemical compositions of siRNA-NS. The size and surface charge of siRNA-NS were measured using Malvern Zetasizer Nano-ZS90 and analysed with Zetasizer software (Malvern Instruments). Each sample was prepared immediately before use and diluted with nuclease-free water. All measurements were carried out at 25 °C, and three measurements with at least 10 sub-runs were performed for each sample.

### siRNA generation from siRNA-NS

For siRNA generation from siRNA-NS, siRNA-NSs were incubated with 0.1 U μl^−1^ of recombinant human Dicer enzyme and reaction buffer (1 mM ATP, 2.5 mM MgCl_2_, 40% Dicer reaction buffer) at 37 °C for 24 h or 48 h. The cleaved products were examined by 20% non-denaturing PAGE in 0.5X Tris-borate-EDTA (TBE) buffer carried out at 120 V for 60 min. Then, the gels were stained with 1X GelRed in 0.5X TBE and analysed under UV with Image Lab software (Bio-Rad).

### *In vitro* gene silencing assay

HeLa-GFP cells were grown in DMEM supplemented with 10% FBS, 100 U ml^−1^ of penicillin, 100 μg ml^−1^ of streptomycin and 1% Antibiotic-Antimycotic at 37 °C in a humidified atmosphere supplemented with 5% CO_2_. The cells were passaged routinely to maintain exponential growth. One day prior to transfection (~90% confluence), the cells were trypsinized, diluted with fresh medium and transferred to 96-well plates (7000 cells per well). The siRNA-NSs were covered with the transfection reagent prior to transfection according to the manufacturer’s instruction, then the cells were treated with the covered siRNA-NS for 24 or 48 h. For cytometry analysis, HeLa-GFP cells were seeded at 24-well plates at the density of 50000 cells per well. The cells were treated with 500 pM of the covered siRNA-NS for 12 h or 24 h. The cells were first stained with Hoechst33342 and propidium iodide, then analysed with NucleoCounter NC-3000 (ChemoMetec).

## Additional Information

**How to cite this article**: Kim, H. *et al*. Generation of siRNA Nanosheets for Efficient RNA Interference. *Sci. Rep*. **6**, 25146; doi: 10.1038/srep25146 (2016).

## Supplementary Material

Supplementary Information

## Figures and Tables

**Table 1:** DNA sequence designs for the fabrication of siRNA-NS.

DNA strands	Sequences	Length (nt)
Linear DNA for anti-GFP siRNA sense strand	5′- Phosphate - ATA GTG AGT CGT ATT AAA AAC TTC AGG GTC AGC TTG CTT GCT GGA TGA AGG ACG GTC GAA CGC AAA ACT TCA GGG TCA GCT TGC TTA TCC CT- 3′	92
Linear DNA for anti-GFP siRNA antisense strand	5′- Phosphate -ATA GTG AGT CGT ATT AAA GCA AGC TGA CCC TGA AGT TTT CTT AGG CTG GAC AAC AAC CAT CTA AAG CAA GCT GAC CCT GAA GTT TTA TCC CT- 3′	92
Linear DNA for scrambled siRNA sense strand	5′- Phosphate - ATA GTG AGT CGT ATT AAA ATG TGA ATG CAG ACC AAA GAA TTG CTG GAT GAA GGA CGG TCG AAA ATG TGA ATG CAG ACC AAA GAA TTA TCC CT -3′	92
Linear DNA for scrambled siRNA antisense strand	5′- Phosphate -ATA GTG AGT CGT ATT AAA TTC TTT GGT CTG CAT TCA CAT TTG GCT GGA CAA CAA CCA TCT AAA TTC TTT GGT CTG CAT TCA CAT TTA TCC CT-3′	92
Primer DNA	5′-TAA TAC GAC TCA CTA TAG GGA T-3′	22

DNA sequences contain the region complementary to primer DNA to form T7 promoter region, sense and anti-sense regions to form siRNAs.
