# Supplementary material for: Generation of siRNA Nanosheets for Efficient RNA Interference
Source: Sci Rep. 2016 Apr 28;6:25146. doi: 10.1038/srep25146 (PMC4848498; doi:10.1038/srep25146)
Supplement: Supplementary Information [file srep25146-s1.pdf]

Supplementary Information

Generation of siRNA Nanosheets for Efficient RNA Interference

Hyejin Kim, Jae Sung Lee and Jong Bum Lee*

Department of Chemical Engineering, University of Seoul, Seoul 02504, Korea

*Fax: +82-2-6490-2364; Tel: +82-2-6490-2372; E-mail: jblee@uos.ac.kr

Correspondence and requests for materials should be addressed to J.B.L.

Supplementary Methods

Analysis of biological stability

Biological stability of siRNA-NS and naked siRNA (23 bp) were analyzed. Both were incubated with nuclease-containing serum for 10 min or 60 min at 37℃, and RNasin® Plus RNase inhibitor (0.5 U μl^{-1}, Promega) was added in order to inactivate serum nuclease-mediated RNA digestion. Then, the reaction solution was analyzed by gel electrophoresis carried out on a 3% agarose gel at 95 V for 80 min. To analyze stability of the naked siRNA and siRNA-NS towards serum, the gel electrophoresis result was processed with Image Lab software (Bio-Rad).

Long-term gene silencing effect

HeLa-GFP cells were seeded at 24-well plate at the density of 50000 cells per well, and treated with 500 pM of anti-GFP siRNA-NS after 24 h. The medium was changed to fresh DMEM in every other day for 6 days. After 6 days, the cells were trypsinized and stained with Hoechst33342 and propidium iodide, then analysed with NucleoCounter NC-3000 (Chemometec) to evaluate long-term gene silencing effect.

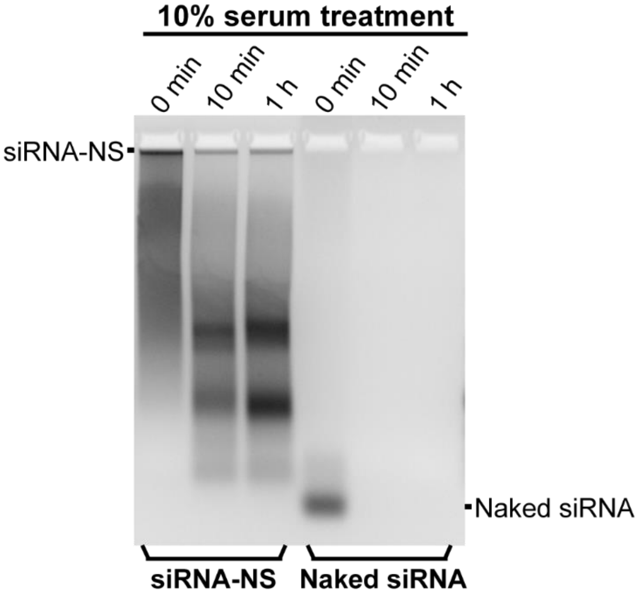

Figure S1. Biological stability of siRNA-NS (left) compared to naked siRNA (right). siRNA-NS (lanes 1-3) and naked siRNAs (lanes 4-6) were treated with 10% FBS for 10 min (lanes 2 & 5) or 1 h (lanes 3 & 6) at 37 ℃, then gel electrophoresis was carried out on 3% agarose gel. The siRNA-NSs were detected within wells, and over 30% of siRNA-NS was able to wit hstand serum nuclease-mediated degradation even after 1 h, while naked siRNA was degraded to undetectable level within 10 min.

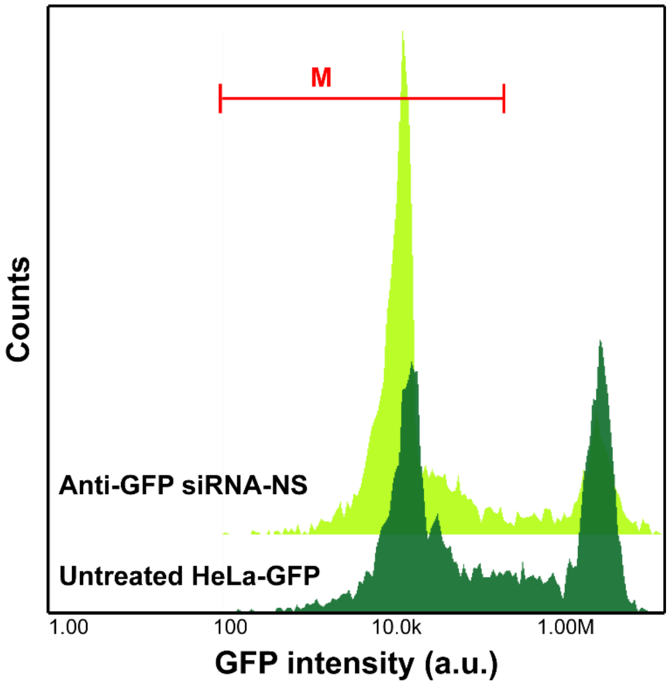

Figure S2. Histograms of GFP intensities of HeLa-GFP cells after treated with 500 pM of anti-GFP siRNA-NS (light green) or culture media only (dark green) for 6 days. Cells with low GFP signals are marked with M, and the percentage of cells within the region M was higher by over 20% for anti-GFP siRNA-NS treated cells, compared to the untreated cells. This result indicates the capability of long-term gene silencing effect of siRNA-NS.
